# Supplementary material for: Clinical transplantation using negative pressure ventilation ex situ lung perfusion with extended criteria donor lungs
Source: Nat Commun. 2020 Nov 13;11:5765. doi: 10.1038/s41467-020-19581-4 (PMC7666579; doi:10.1038/s41467-020-19581-4)
Supplement: Supplementary file 1 — Supplementary Information [file 41467_2020_19581_MOESM1_ESM.pdf]

Clinical transplantation using negative pressure ventilation ex situ lung perfusion with extended criteria donor lungs

Max T Buchko, Nasim Boroumand, Jeffrey C Cheng, Alim Hirji, Kieran Halloran, Darren H Freed and Jayan Nagendran.

Supplementary Information

Supplementary Table 1-4

Supplementary Note 1

Supplementary Table 1. Donor inclusion and exclusion criteria.

Donor Inclusion Criteria (any of the following):

1. P:F ratio < 300mmHg
2. Maastricht III or IV deceased from cardiac death donors (DCD)
3. Greater than 10 units of blood transfusion
4. Expected cold ischemic time > 6 hours
5. Donor age > 55 years old
6. Pulmonary edema, defined as bilateral interstitial infiltrates without evidence of infection, detected on the last chest radiograph by the lung-transplantation physician assessing the donor
7. Poor lung deflation or inflation during direct intraoperative visual examination at the donor site

Donor exclusion criteria (any of the following):

1. Established pneumonia
2. Severe mechanical lung injury (i.e., contusions in more than one lobe) or trauma determined by chest x-ray, bronchoscopy, CT scan or visual inspection
3. Gross gastric aspiration within the lungs
4. Donor lungs have active infectious disease such as HIV, Hepatitis B, Hepatitis C, West Nile Virus (WNV), HTLV, or Syphilis (if this information not available at start of EVLP, it should be re-assessed prior to transplant)

Recipient inclusion criteria (all of the following):

1. Patients aged >18 years old
2. Accepted on the transplant institute’s waitlist for bilateral transplantation
3. Provided prospective written consent to being part of the trial

Recipient exclusion criteria (any of the following):

1. Multi-organ recipients or re-transplants

2. Hemodialysis or severe chronic renal dysfunction
3. HIV, Hepatitis, or other infection that excludes subject from transplant in the study
4. Concurrent cardiac procedure
5. Pre-operative mechanical circulatory support or mechanical ventilation (excluding CPAP or BiPAP)

P:F Ratio, Ratio of PaO2:fraction of inspired oxygen, HIV, human immunodeficiency virus, HTLV, human T-lymphotropic virus, CPAP, continuous positive airway pressure non-invasive ventilation, BiPAP, bilevel positive airway pressure non-invasive ventilation

Supplementary Table 2. Donor Eurotransplant ECD Characteristics.*

Donor Characteristics	Trial Patients (n=12)
Age (yrs)	43 ± 3
Donor History** – n (%)	
Compromised	4 (33)
Uncompromised	8 (67)
Smoking History*** - n (%)	7 (58)
Chest radiograph – n (%)	3 (25)
Clear	10 (83)
Consolidation	2 (17)
Bronchoscopy	1 (8)
Clear	11 (92)
Purulent	1 (8)
Donor P:F Ratio (mmHg)	234 ± 38
Eurotransplant ECD Donor Score	9.6 ± 0.6

ECD, extended criteria donor, P:F Ratio, Ratio of PaO2:fraction of inspired oxygen.

* Plus–minus values represent mean ±SE

**Compromised donors include a history of prior malignancy, sepsis, drug abuse, meningitis, or positive virology status (HBsAg,HBcAb, HCVAb, and anti-CMV).

***Smoking history greater than 10 pack years.

Supplementary Table 3. Graft ischemic and ESLP times. All times are in minutes.

Donor	Cold ischemic time prior to ESLP	Warm ischemic time if DCD	Time on ESLP	Cold ischemic time following ESLP – Right Lung	Cold ischemic time following ESLP – Left Lung	Total time to implantation – Right Lung	Total time to implantation – Left Lung
1	278	N/A	174	184	87	636	539
2	252	N/A	229	65	151	546	632
3	218	23	217	163	69	621	527
4	124	N/A	185	154	73	463	382
5	193	N/A	143	180	180	516	516
6	235	N/A	143	179	91	557	469
7	84	N/A	159	122	69	365	312
8	120	N/A	135	114	43	369	298
9	268	14	179	76	185	537	646
10	238	N/A	162	207	100	607	500
11	225	N/A	201	178	81	604	507
12	251	15	261	208	84	735	611

N/A, not applicable, ESLP, ex-situ lung perfusion, DCD, donation after circulatory death.

Supplementary Table 4. Contemporary cohort of recipients receiving lung transplantation without ESLP (n=57).

Mean Age (years)	52 ± 16
Recipient Etiology (%)	
Interstitial Lung Disease	11
Emphysema	34
Cystic Fibrosis	11
PAH	6
Pulmonary Fibrosis	9
Talcosis	7
Other	22
Median Mechanical Ventilation Time (hours)	42
Median ICU LOS (days)	6
PGD 3 at 72 hours (%)	15
Survival to 30 days (%)	99
Survival to 1 year (%)	93

Supplementary Note 1. Study Protocol.

The University of Alberta Negative Pressure Ventilation Ex-Vivo Lung Perfusion (NPV-EVLP) Trial

Protocol Number: Pro00070552

Version Date: 23-Jan-2018

Study Sponsor:

University of Alberta

Faculty of Medicine and Dentistry

Department of Surgery

2D, Walter C. Mackenzie Health Sciences Centre

8440 - 112 Street

Edmonton, AB

Canada, T6G 2B7

Study Synopsis

**Title**	**The University of Alberta Negative Pressure Ventilation Ex-Vivo Lung Perfusion (NPV-EVLP) Trial**
**Short Title**	UA NPV-EVLP
**Protocol Number**	Pro00070552
**Phase**	Proof of Concept, Early Feasibility, Phase I
**Methodology**	Non-Randomized; Interventional; Prospective; Device
**Study Duration**	We intend to have our first patient transplanted by Fall 2017, with an approximate study enrollment end date of December 31, 2018.
**Study Center(s)**	This is a single centered trial with trial transplantations taking place at the University of Alberta Hospital/Mazankowski Alberta Heart Institute.
**Objectives**	The objective of this study is to show safety of a University of Alberta developed device for the purpose of evaluation of lung function ex-vivo to determine organ suitability for transplantation.
**Number of Subjects**	A maximum of 12 patients will be enrolled in this study.
**Diagnosis and Main Inclusion Criteria**	Inclusion into the study will be the offer of donor lungs for transplantation that do not meet the criteria of a standard donor, and as such are deemed to be marginal in donors >18 years of age. Recipients will be adults who are listed for a double lung transplant meeting the studies eligibility criteria who have provided written study consent.
**Study Product**	The University of Alberta generated Negative Pressure Ventilated Ex-Vivo Lung Perfusion (NPV-EVLP) device.
**Duration of Administration**	Lungs will be perfused for 2-6 hours on the device to determine suitability for transplantation.
**Reference Therapy**	There is no reference therapy, as donor lungs deemed marginal would otherwise not be transplanted.
**Statistical Methodology**	Descriptive/raw data will be captured and presented.

Table of Contents

1 BACKGROUND ... 11

1.1 INTRODUCTION ... 11
1.2 BRIEF HISTORY ... 11
1.3 INVESTIGATIONAL DEVICE ... 12
1.4 PRECLINICAL ANIMAL DATA ... 12

2 RISKS AND BENEFITS ... 13

2.1 POTENTIAL RISKS ... 13
2.2 RISK MINIMIZATION ... 13
2.3 POTENTIAL BENEFITS ... 14
2.4 TRIAL CONDUCT ... 14

3 TRIAL OBJECTIVES ... 14

3.1 PRIMARY ENDPOINTS ... 14
3.2 SECONDARY ENDPOINTS ... 15
3.3 SAFETY ENDPOINTS ... 15

4 TRIAL DESIGN ... 15

4.1 TRIAL TREATMENT AND ENROLLMENT ... 16
4.2 DURATION OF SUBJECT FOLLOW-UP ... 16
4.3 DATA IDENTIFICATION ... 17
4.4 DISCONTINUATION ... 18
4.5 DONOR LUNG USAGE ... 19
4.6 BIOPSY AND PATHOLOGICAL ASSESSMENTS ... 19
4.7 DEVICE ACCOUNTABILITY ... 19

5 STUDY POPULATION ... 19

5.1 DONOR/RECIPIENT DEMOGRAPHICS AND CHARACTERISTICS ... 19
5.2 PRE-NPV-EVLP DONOR ELIGIBILITY CRITERIA ... 20
5.2.1 Donor MUST meet ANY ONE of the following Inclusion Criteria to proceed with NPV-EVLP: ... 20
5.2.2 Donor Exclusion Criteria to NOT proceed with NPV-EVLP: ... 20
5.3 POST-NPV-EVLP DONOR ELIGIBILITY CRITERIA ... 20
5.3.1 Donor Inclusion Criteria to proceed with Transplant: ... 20
5.3.2 Donor Exclusion Criteria to proceed with Transplant: ... 20
5.4 RECIPIENT ELIGIBILITY CRITERIA ... 21
5.4.1 Recipient Inclusion Criteria ... 21
5.4.2 Recipient Exclusion Criteria ... 21
5.5 SUBJECT WITHDRAWAL ... 21
5.6 TREATMENT OF SUBJECTS ... 21

6 ASSESSMENT & REPORTING OF SAFETY PARAMETERS ... 22

6.1 RECIPIENT SAFETY ENDPOINTS ... 22
6.2 ADVERSE EVENT RECORDING AND REPORTING ... 22
6.3 ABNORMAL CLINICAL & LABORATORY RESULTS ... 23
6.4 ADVERSE EVENT DEFINITIONS ... 23
6.5 SERIOUS ADVERSE EVENTS ... 24

7 STATISTICAL PLAN ... 25

7.1 STATISTICAL METHODS ... 25
7.2 SUBJECT POPULATION(S) FOR ANALYSIS ... 25

7.3 SIGNIFICANCE 26
7.4 TERMINATION CRITERIA 26

8 DIRECT ACCESS TO SOURCE DATA/DOCUMENTATION 27

9 TRAINING, DEVIATIONS & MONITORING 27

9.1 STUDY STAFF TRAINING 27
9.1.1 Procurement of Donor Lungs 27
9.1.2 Informed Consent at Time of Organ Offer 28
9.1.3 Instrumentation and Perfusion of Donor Lungs 28
9.2 PROTOCOL DEVIATIONS 28
9.3 STUDY MONITORING 29

10 ETHICAL CONSIDERATIONS 29

11 DATA HANDLING AND RECORD KEEPING 29

11.1 COMPLETING, SIGNING AND ARCHIVING CASE REPORT FORMS 29
11.2 DATA MANAGEMENT AND ARCHIVING 30

12 ABBREVIATIONS & DEFINITIONS 30

13 REFERENCES 31

14 APPENDICES .... ERROR! BOOKMARK NOT DEFINED.

1 Background

1.1 Introduction

This project is focused on helping one of the most vulnerable patient populations in medicine, patients with end-stage chronic lung disease[1]. Lung transplantation is the only cure for end-stage lung disease, however, due to the persistent shortage of donor organs, either due to low organ donation rates or unacceptable organs, only a minority of patients receive desperately needed lung transplants. Currently less than 30% of potential donated thoracic organs are being used for transplantation. The major causes for under-utilization of donor thoracic organs are injury sustained by the lungs in trauma or emergency resuscitation or lungs that come from donors who are pronounced dead due to cardiac arrest (known as DCD donors)[2]. It has been hypothesized that these injuries may be reversible or repairable if there was an opportunity to evaluate and repair these organs outside of the body (ex-vivo), prior to transplantation. In fact, studies have shown that the use of normothermic Ex-Vivo Lung Perfusion (EVLP) has increased the rate of donor organ utilization at centers that have adopted the technology[7].

Current methodology for all clinically available EVLP devices uses Positive Pressure Ventilation (PPV). Researchers at the University of Alberta (UofA), however, have developed an EVLP device that will apply Negative Pressure Ventilation (NPV) to the lungs, as opposed to PPV, which is the most ideal mimicry of native lung physiology. The objective of this early feasibility safety trial is to show that the UofA developed NPV-EVLP device is acceptable in evaluating and improving the quality of marginal donor lungs compared to currently used EVLP devices, ultimately allowing for these types of donor lungs to be safely transplanted into patients on the lung transplant recipient waitlist.

1.2 Brief History

The first successful human single lung transplantation occurred in 1983 and the first double lung transplantation in 1986 in Toronto[3, 4]. The use of normothermic ex-vivo lung perfusion was pioneered by Stig Steen and colleagues, from Sweden, in 2001[5]. This technology was created primarily to aid in the start of transplanting lungs from DCD donors, nonetheless this technology can be extended to all donor lungs for objective assessment ex-vivo. Since the original utilization of the ex-vivo technology, rigorous methodological evaluation has occurred which has improved the efficacy of the device. The original EVLP was only stable for two hours, while today's EVLP has been shown to be stable for up to 12 hours. The majority of this advancement has been performed by careful pre-clinical and clinical experiments by Shaf Keshavjee and the thoracic transplant program at Toronto General Hospital[6].

In 2011, the same Toronto team performed the first clinical trial using normothermic ex-vivo lung perfusion in human transplantation[7]. Ex-vivo lung perfusion was performed for 4 hours in 23 'high-risk' marginal donor lungs that were deemed not suitable for transplantation. Of the 23, 20 donor lungs were assessed and reconditioned to acceptable criteria and went on to be clinically transplanted. This study concluded that ex-vivo lung perfusion was safe in a clinical setting. Two important conclusions from this work were, firstly, that it showed marginal donors with lower oxygenation challenge tests can be placed on the EVLP system and undergo perfusion for 4 hours safely. Lungs shown to be stable with

acceptable gas exchange and dynamic airway compliance were transplanted with acceptable clinical outcomes. Secondly, it showed that the DCD donor could be evaluated on this device objectively for 4 hours to evaluate the quality of donor lungs prior to transplantation. This significant advance in donor procurement for lung transplantation opens the opportunity for further improvement.

1.3 Investigational Device

The Negative Pressure Ventilated Ex-Vivo Lung Perfusion (NPV-EVLP) is an automated platform designed to maintain donor lungs in an environment most mimicking the human body at normothermia with vascular perfusion and ventilatory gas exchange. This platform will allow for physiological assessment of donor lungs to inform clinical decisions regarding usability of marginal donor lungs for transplantation. The NPV-EVLP consists of a negative pressure chamber, a perfusion pump, integrated software for automation, and disposable perfusion sets.

1.4 Preclinical Animal Data

Background: Normothermic Ex-Vivo Lung Perfusion (EVLP) has increased the rate of donor organ utilization, and increased volumes of lung transplantation at centers that have adopted the technology. Current methodology for EVLP uses Positive Pressure Ventilation (PPV) with all clinically available devices. As ideal mimicry of native lung physiology would apply a negative pressure to the pleural surface of the lung (Negative Pressure Ventilation, NPV), we hypothesize that NPV would be superior to PPV during EVLP.

Methods: A fully automated NPV EVLP platform was developed and compared, pig and human, lungs perfused by conventional (PPV) EVLP. All lungs were perfused for 12 hours and physiologic parameters, cytokine profile, bronchopleural fistula (BPF) and edema formation were analyzed. A total of 24 pig lungs were perfused, divided equally into 4 groups based on ventilation strategy and perfusate composition: acellular (STEEN solution™) and cellular (packed Red Blood Cells + STEEN solution™). Preliminary unutilized human lungs compared Cellular-NPV (N=2) and Cellular-PPV (N=2).

Results: Using ANOVA pairwise comparison (mean±SE), pig and human lungs stable trends, in lung oxygenation (>400 mmHg) and physiological parameters. Cytokine analysis of the pig lungs showed significantly lower TNFa, IL-6, and IL-8 production with an NPV strategy regardless of perfusate ($P<0.05$). Moreover, there was a 38% lower incidence of BPF with a NPV vs. PPV strategy ($P=0.02$). Edema after 12 hours of EVLP for pRBCs using NPV and PPV were 15.4±2.0% and 40.6±9.0% ($P<0.05$); while with acellular using NPV and PPV was 33.2±6.4% and 88.1±11.0%, respectively. On the other hand, with human lung perfusion we interestingly found edema at 12 hours for pRBCs using NPV and PPV were -10.8±9.0% and 37.1±9.0% ($P<0.05$), respectively.

Conclusions: Negative pressure ventilation (NPV) is potentially more beneficial compared to traditional positive pressure ventilation (PPV), with significantly less inflammation, bullae, and edema formation during extended EVLP for both perfusate groups. The value of a NPV strategy may lead to further improvements to currently available clinical EVLP platforms.

2 Risks and Benefits

2.1 *Potential Risks*

There are inherent risks associated with primary lung transplants. Potential risks associated with this type of procedure include post-operative complications, such as: infection, compromised kidney function, diabetes, high blood pressure, cancer, neurological complications, graft rejection, graft vessel disease (representative of chronic rejection) and possibly death. The overall 30-day and 1-year survival rates for patients receiving lung transplants at the UAH from 1986 to 2015 are 96% and 86% respectively, which would be the expected survival of patients enrolled in the trial.

The potential risks associated with the device would be causing damage to donor lungs that would make them unusable for transplantation. Since the only lungs being evaluated in this trial will be lungs already deemed marginal by standard criteria, the lungs are already deemed unusable for transplantation. The only potential for transplantation is if the device helps improve the quality of the donor lungs based on physiologic lung ventilation and perfusion parameters that are also correlated with perfusate oxygenation.

2.2 *Risk Minimization*

The investigators of this trial have recruited numerous means, including the novel design of the ex-vivo device, risk analysis and preclinical testing, and the clinical protocol itself, to minimize the risks to subjects and to protect their safety and welfare. The clinical protocol incorporates multiple procedures to minimize the risks to patients such that the benefits far outweigh the potential risks. Patients will be monitored pre-, intra- and post-operatively to ensure safety. Drs. Freed and Nagendran have performed over 100 lung transplant surgeries and lead the University of Alberta clinical lung transplant program for use of marginal donor lungs. The two investigators have also performed a combined 26 clinical ex-vivo lung perfusions using other investigational devices from ex-vivo perfusion device companies with successful transplantation of 25 sets of lungs. Dr. Freed has built the device, as such understands all aspects of its function completely. Patients in the study will have thorough follow-up with frequent monitoring visits along with routine clinical protocols as per the University of Alberta Lung Transplant program to detect adverse events and provide therapy as indicated. The study will be monitored on an ongoing basis by the University of Alberta's Quality Management in Clinical Research (QMCR) to verify identification, documentation and analysis of adverse event, compliance with the study protocol, and assessment of patient safety at all times within the study. An independent Data Safety Monitoring Board (DSMB) will be established by the University of Alberta's QMCR to provide oversight on the progress of the trial and the safety data and endpoints.

Prior to its first clinical use in this trial, the NPV-EVLP device hardware console will have undergone a Canadian Standards Association (CSA) Special Inspection to relevant standards, as well as inspection and approval by the UAH Clinical Engineering department. Sterile disposable perfusion elements will be a combination of off-the-shelf commercial medical products (sterile tubing, filters, etc.) and a custom plastic organ chamber that has undergone sterility validation (per ISO 11135:2014) and biocompatibility

testing (per ISO 10993) in accordance with applicable International Organization of Standardization (ISO) standards.

2.3 Potential Benefits

The benefits derived from using the intended device is that lungs that were deemed marginal and unacceptable for routine transplantation will have an opportunity to be assessed by the NPV-EVLP to determine if they are indeed suitable for transplantation based on standardized criteria currently accepted in the lung transplant community when Ex-Vivo Lung Perfusion assessments are performed. The deliverable at the end of the study will be a 10% increase in donor utilization from 30% to 40%. This will translate to a 50% decrease in the lung transplant wait list mortality. The technology will lead to a great geographic expansion of where donors can be accepted from, and allow us to use potential injured organs. Therefore it increases our donor utilization and availability without actually increasing the number of donors. In short, this project truly has the potential of revolutionizing the way thoracic transplantation is performed in this country. It is a truly translational project as this novel NPV-EVLP device was developed at the U of A with testing in animals to now deliver a first in human safety trial in 2.5 years. It is indeed a very novel and needed research endeavour to save lives of vulnerable Canadians with end-stage chronic lung disease.

2.4 Trial Conduct

This study will be conducted in compliance with the protocol approved by the University of Alberta Health Research Ethics Board (HREB), Good Clinical Practice (E6) guidelines, TCPS2 and applicable University of Alberta policies and procedures. No amendments to the protocol will be implemented without the prior review and approval of the HREB except where it may be necessary to eliminate an immediate hazard to a research subject. In such case, the deviation will be reported to the HREB as soon as possible.

3 Trial Objectives

The purpose of this Phase I Early Feasibility Proof of Concept clinical trial will be to evaluate initial performance and safety of the NPV-EVLP device to assess and improve the function of marginal donor lungs. By nature, efficacy measures and outcomes of the device will also become evident from the results of this study. Any efficacy data from this early feasibility trial will be captured and used to guide the development of subsequent Phase I, II and III trials.

All endpoints and outcomes will be compared between the experimental group (those recipients who receive lungs post NPV-EVLP) versus a contemporaneous group of recipients (those who receive lungs in the conventional/Standard of Care (SOC) method).

3.1 Primary Endpoints

The primary end point is a co-primary endpoint comparing patient survival rates post transplantation at Day30 and rates of Primary Graft Dysfunction (PGD) Grade 3 (Table 1) in the first 72 hours with success measured only if both endpoints are met.

**Table 1: International Society for Heart and Lung Transplantation Primary Graft Dysfunction (PGD) Grading Schema** (Adapted from Christie et al., 2005)		
**Grade**	**PaO_2/FiO_2 (mmHg)**	**Radiographic Infiltrates Consistent with Pulmonary Edema**
0	>300	Absent
1	>300	Present
2	200-300	Present or Absent
3	<200	Present or Absent
**Note 1**: This grading system will be applied at T0 (time of ICU admission), T24, T48, and T72 post-transplant time points (or closest to these time points) per ISHLT guidelines. **Note 2**: Patients who are no longer on a mechanical ventilator at time of PDG assessment can be classified as Grade 0 or 1 ONLY based on chest X-ray findings.		

3.2 Secondary Endpoints

- PGD scores at T0, T24, T48, and T72Hrs
- Intensive Care Unit Length of Stay
- Hospital Length of Stay
- Duration of Invasive Mechanical Ventilation post-transplantation
- FEV1 at 6Mos and 1Yr
- Quality of Life measured by functional status, physical capability and employment limitations at 6Mos and 1Yr

3.3 Safety Endpoints

Safety endpoints include the number of lung-related serious adverse events (SAEs) through to the Day30 follow-up after transplantation (T0) per subject. This endpoint will be defined to consist of the following serious adverse events that are defined further in Section 6.1:

- Acute rejection
- Respiratory failure
- Bronchial anastomotic complication
- Major pulmonary-related infection

4 Trial Design

This is a prospective, non-randomized, interventional trial, taking place solely at the University of Alberta Hospital/Mazankowski Alberta Heart Institute. Lungs deemed marginal, based on standard lung donor criteria that meet the study's eligibility criteria, will be assessed on our NPV-EVLP device to determine suitability for lung transplantation. Objective assessment of quality will be made while the

lungs are on the device based on pre-determined functional parameters of lung physiology. Once a total of 12 sets of lungs are transplanted after using the device, safety will be determined by post-operative lung function and recipient survival.

4.1 Trial Treatment and Enrollment

We plan on applying a graduated enrollment strategy in this study. Once we have enrolled our first participant (ie. lung transplant receive under this protocol), we will not enroll another patient until a period of one month post procedure to allow the investigators to assess primary outcomes (Survival at Day30 and PGD Grades in the first 72hours) in our first case. This will give the investigators an opportunity to evaluate the technology and it's performance prior to proceeding with our second case.

After initial screening, appropriately obtained informed consent and confirmation of eligibility at time of transplant, those recipients (a total of 12 subjects) who agree to continue as participants will receive reconditioned marginal lungs should the lungs on the device meet acceptable criteria to proceed with clinical transplantation. These subjects will form the experimental group. We will compare the outcomes, trial and safety endpoints of the experimental group to those patients who undergo conventional/SOC lung transplantation during the same study period. Consenting will not be performed for SOC/conventional transplants as data that will be provided to us by the Transplant Services program will be de-identified and in aggregate format with only essential outcome data to perform a comparison analysis.

Lungs deemed marginal based on standard lung donor criteria that meet study eligibility will be physiologically assessed during ex-vivo perfusion with Steen Solution, 3 units of packed red blood cells (pRBCs) and 500mL of 5% albumin. NPV-EVLP of these lungs will be performed with the addition of Nitroglycerin (50mg), $NaHCO3$ (40mEq), Methylprednisolone (500mg), unfractionated Heparin (40,000 units), Cefazolin (1000mg), Voriconazole (200mg), and Ciprofloxacin (400mg). With respect to the decision of lung utilization post-EVLP, eligibility criteria listed in Section 5.3 will need to be met. Lungs will also be excluded if they are deemed unsuitable based on the clinical judgment of the lung transplant surgeon.

4.2 Duration of Subject Follow-Up

The study participants will be closely followed from their transplant date until they are discharged by the post-transplant team as well as the study PI/team to capture primary endpoint data and any (serious) adverse events. Study subjects will be monitored through routine clinic visits or via medical records review at Day30 to collect survival status. Subjects will be followed up for 1 year post-operatively in conjunction with their routine post-transplant clinic visits with the Transplant Services program. There will be follow-up quality of life questionnaires and spirometry performed at 6 months and 1 year both of which are SOC post-transplant activities). All data collection and protocol specified assessments will be done per Tables 2 and 3 at time points specified.

4.3 Data Identification

The tables below represent the schedule of assessments and evaluations to be collected for the donors and the recipients. There are no study specific tests/exams/investigations that will be performed. Only available SOC data collection will be required for this study.

Table 2: Donor Time and Events Schedule

Assessment	Screening & Acceptance	NPV-EVLP Preservation
Donor Organ ID	X	
Eligibility Pre-EVLP (per Section 5.2)	X	
Demographics/Characteristics*	X	
Donor Cause/Declaration of Death*	X	
Donor Medical/Social/Admission History*	X	
Final Blood Gas & PaO_2/FiO_2 Ratio	X	
Ventilation Settings at Final Blood Gas*	X	
Hemodynamics at Final Blood Gas*	X	
Final Chest X-ray Findings*	X	
Final Bronchoscopy Findings*	X	
Organ Retrieval, Operative & Perfusion Details		X
NPV-EVLP Pre-Instrumentation Blood Gas		X
NPV-EVLP Instrumentation Details		X
NPV-EVLP Monitoring Observations & Blood Gases		X
NPV-EVLP Preservation Parameters		X
NPV-EVLP Final Blood Gas		
Eligibility Post-EVLP (per Section 5.3)		X
Reason for Non-Transplanting Lungs		X

Footnotes: *Collect if available or done per SOC.

Table 3: Recipient Time and Events Schedule

Assessment	Pre-Tx	T0[1]	T24[2]	T48[2]	T72[2]	Day7[3]	D/C[4]	Day30[5]	6Mos[6]	1Yr[6]
Informed Consent	X									
Eligibility (per Section 5.4)	X									
Demographic/Characteristics*	X									
History/Lifestyle Factors*	X									
Medical/Surgical History*	X									
Pulmonary Assessments*	X									
Transplant Details		X								
Graft Surveillance		X	X	X	X					
PGD Scores		X	X	X	X					
Bronchoscopy	X					X	X		X	X
Patient Survival		X	X	X	X			X	X	X
Graft Survival		X	X	X	X			X	X	X
Chest X-Ray*	X	X	X	X	X					
Hemodynamics*	X	X	X	X	X		X			
Mechanical Circulatory Support	X	X	X	X	X		X			
Invasive Ventilator Support		X	X	X	X		X			
ICU LOS (including Re-Admissions on Index Hospitalization)							X			
Hospital LOS (including Re-Admissions)							X			
(Serious) Adverse Events		X	X	X	X		X	X		
Lung Related SAEs (per Section 3.3)		X	X	X	X		X	X		
Pulmonary Function Test	X						X		X	X
Quality of Life Measurements									X	X

Footnotes: ***Collect if available or done per SOC; **1**: T0 = time of ICU admission +4Hrs; **2**: T24, 48, 72 ±4Hrs; **3**: Day7 ±1Day; **4**: D/C (Lat Prior to D/C); **5**: Day30 ±10Days; **6**: 6Mos and 1Yr ±60Days.

4.4 Discontinuation

After donor lungs have been perfused and evaluated on our NPV-EVLP device and are deemed not suitable for transplantation (i.e. donor lung suitability for transplantation after 2-6hours of EVLP is not met (see Section 5.3), the potential recipient of those lungs will be notified that the lungs are not

acceptable for transplantation and they will not be transplanted. The recipient will be returned to the same level of priority on the lung transplant waitlist thereafter.

4.5 Donor Lung Usage

We anticipate that 15 sets of marginal donor lungs will be perfused to attain 12 sets of lungs that meet study eligibility and transplant suitability that will go on to be transplanted into 12 recipients. This assumes a conversion failure rate of 25%. All marginal donor lung outcomes (successes and failures) will be captured in a "Donor Lung Usage Log" (Appendix A).

4.6 Biopsy and Pathological Assessments

We plan on taking two small peripheral wedge biopsies (approximately $1cm^3$) from the donor lungs lingual pre- and post-EVLP which will be used for standard formalin-fixed paraffin-embedded (FFPE) tissue processing. We will use the FFPE biopsy samples to perform the following pathological assessments: histology and gene expression. We hope to use the data gathered from these assessments to develop a new test that will measure the activity of specific damage-related genes in donor lungs. Based on our previous porcine animal model work (using the same methodology), we anticipate the expression of known acute lung injury genes to be decreased and repair-related genes to be increased with EVLP. Correlating these gene expression changes with function, histology and clinical outcomes will allow us to develop a clinically-meaningful molecular test for monitoring donor lung damage and repair in a more precise, objective and mechanistic manner. This will allow us to further develop and optimize EVLP so it can be as effective as possible, which will ultimately allow for more potential donor lungs to be used for transplantation and for more lung disease patients to be provided with life-saving transplant surgery.

4.7 Device Accountability

There will be one NPV-EVLP device and at minimum 12 single use organ chambers in the Mazankowski Alberta Heart Institute Level 3 OR awaiting a donor organ offer. The NPV-EVLP device will have a designated manufacturer, model and serial number. It will also have a CSA Special Inspection Sticker and a label specifying voltage, amperage and frequency. Each organ chamber will have a serial number associated with it and an expiry date. All of these items will be recorded on a "Device Accountability Log" along with date of receipt and date of use. The shipment records and accountability logs will be kept in the study regulatory binder.

5 Study Population

5.1 Donor/Recipient Demographics and Characteristics

The study (recipient) population will consist of adult patients (>18yrs) listed on the recipient waitlist at the University of Alberta Lung Transplant Program who have consented to participate in this study and meet all recipient eligibility criteria.

The population of donor lungs will be any donor lungs that are offered to our institution for transplantation, but deemed marginal by standard donor lung assessment criteria. These donors will

have, prior to their expiration, consented, or their family member had consented through their respective Organ Donor Office (ODO), to donate their organs. These marginal donor lungs have to meet strict pre-NPV-EVLP eligibility criteria (Section 5.2) to proceed with ex-vivo perfusion, as well as post-NPV-EVLP eligibility criteria (Section 5.3) to proceed with transplantation.

5.2 PRE-NPV-EVLP Donor Eligibility Criteria

5.2.1 Donor <u>MUST</u> meet ANY ONE of the following Inclusion Criteria to proceed with NPV-EVLP:

a) Best ratio of the PaO_2 to FiO_2 of < 300mmHg;
b) Pulmonary edema, defined as bilateral interstitial infiltrates without evidence of infection, detected on the last chest radiograph by the lung-transplantation physician assessing the donor;
c) Poor lung deflation or inflation during direct intraoperative visual examination at the donor site;
d) Donor age is ≥ 55 years;
e) Expected cold ischemic time > 6 hours;
f) Blood transfusions ≥ 10 units; or
g) Donation after cardiac death (DCD), as defined by Maastricht category III (donor without a heartbeat and with cardiocirculatory death imminent after withdrawal of treatment) or category IV (cardiocirculatory death in a brain-dead donor).

5.2.2 Donor Exclusion Criteria to <u>NOT</u> proceed with NPV-EVLP:

a) Donor lungs with established pneumonia;
b) Severe mechanical lung injury (i.e., contusions in more than one lobe) or trauma determined by chest x-ray, bronchoscopy, CT scan or visual inspection; or
c) Gross gastric aspiration within the lungs
d) Donor lungs have active infectious disease such as HIV, Hepatitis B, Hepatitis C, West Nile Virus (WNV), HTLV, or Syphilis (if this information not available at start of EVLP, it should be re-assessed prior to transplant).

5.3 POST-NPV-EVLP Donor Eligibility Criteria

5.3.1 Donor Inclusion Criteria to proceed with Transplant:

a) Surgeon must be satisfied with the clinical evaluation and appearance of the lungs; if not, reason for refusal must be documented;
b) Lungs show **PaO_2/FiO_2 ratio ≥ 350mmHg**; **AND**
c) Deterioration of less than 15% from baseline for physiological measurements pulmonary vascular resistance (PVR), dynamic compliance and peak inspiratory pressure.

5.3.2 Donor Exclusion Criteria to proceed with Transplant:

a) Lungs show a PaO_2/FiO_2 ratio of < 350mmHg;
b) Greater than 15% functional deterioration across the following physiological parameters: PVR, dynamic compliance and peak inspiratory pressure;
c) Donor lungs are positive for infectious disease such as HIV, Hepatitis B, Hepatitis C, West Nile Virus (WNV),HTLV, or Syphilis.

5.4 Recipient Eligibility Criteria

5.4.1 Recipient Inclusion Criteria

a) Patients on our institution's waitlist requiring bilateral transplantation
b) Male or Female 18 years of age or older
c) Written informed consent provided.

5.4.2 Recipient Exclusion Criteria

a) Multi-organ recipient or re-transplant
b) HIV, Hepatitis, or other infection that excludes subject from transplant in the study
c) Subject is on hemodialysis or has chronic severe renal dysfunction
d) Concurrent cardiac procedure
e) Recipient is on Nova Lung, ECMO or on mechanical ventilation (CPAP and BiPAP not exclusionary)

5.5 Subject Withdrawal

All subjects are informed, at the time that they sign the study's consent form, that participation in this study is strictly voluntary and that they are free to withdraw from the study at any time without prejudice and without it affecting any of the medical care they are entitled to receive. The participants will also be informed that at any time prior to their lung transplant, they can be withdrawn from the study by the Investigator(s) due to not meeting the study's eligibility criteria, the marginal lungs not being reconditioned to a transplantable state or other reasons concerning the health or wellbeing of the subject. Should a subject decide to withdraw or should the investigator decide to withdraw the subject for any reason, all efforts will be made to complete and report the observations up to the time of withdrawal as thoroughly as possible. Any reasons for withdrawal will be documented on an End of Study CRF and filed in the subject's research records. If a subject decides to withdraw, all data that was collected as part of the research study up to the date of withdrawal of their consent will still be used to protect the quality of the study.

5.6 Treatment of Subjects

Once the subject's eligibility is re-confirmed, the subject will be notified on whether the donor lungs that have been matched to them are a set of marginal donor lungs requiring the assessment and intervention of the NPV-EVLP. If the subject agrees to accept these marginal lungs after they are reconditioned to a transplantable state according to the clinical judgment of the transplant surgeon and study Investigator, then the transplant will go ahead per our institution's standard of care lung transplants. If the subject refuses the reconditioned donor lungs, then he/she will be placed back on the recipient waitlist and the lungs will be offered to the next suitable recipient. If all suitable recipients refuse the donor lungs or the lungs are deemed not suitable for transplantation following EVLP treatment, then the lungs will be discarded per the Edmonton Zone Human Organ Procurement and Exchange policy for discarding organs and tissues.

6 Assessment & Reporting of Safety Parameters

6.1 Recipient Safety Endpoints

The following safety endpoints are lung-related serious adverse events (SAEs) through to the Day30 follow-up after transplantation (T0) per subject. This endpoint will be defined to consist of the following serious adverse events:

- **Acute rejection** – defined as moderate to severe according to the ISHLT PGD grading schema listed in Table 1.
- **Respiratory failure** – defined as an impairment of respiratory function requiring reintubation, tracheostomy or the inability to discontinue invasive ventilatory support within 4 days (96 hours) post-transplant. This excludes re-intubation for re-operation or temporary intubation for diagnostic or other therapeutic procedures.
- **Bronchial anastomotic complication** – defined as moderate to severe necrosis (mucosal and/or extending to bronchial wall) at the bronchial anastomotic site due to ischemic injury, with or without bronchial anastomotic dehiscence (Grade II – IV) as visualized by flexible fiber optic bronchoscopy and/or chest CT (for dehiscence), and/or requiring antibacterial/antifungal treatment, chest tube placement (tube thoracostomy), uncovered metallic stent placement, primary repair, open surgical repair, pneumonectomy, or re-transplantation. Bronchoalveolar (BAL) specimen/culture should be obtained if evidence of focal infection prior to initiating treatment.
- **Major pulmonary-related infection** – defined as a clinical infection of pulmonary origin that is treated with antibacterial/antifungal/antiviral agents (nonprophylactic). This category includes, but is not limited to: bacterial pneumonia/tracheobronchitis (*Pseudomonas aeruginosa*, coagulase-positive and coagulase-negative *Staphylococcus* species (including MRSA), *Chlamydia pneumoniae*, Enterococcus (including VRE), Enterobacteriaceae), Cytomegalo virus (CMV0 pneumonitis/pneumonia, Herpes Simplex Virus (HSV) tracheobronchitis/pneumonitis/pneumonia, Aspergillus tracheobronchitis/pneumonia, and aspiration pneumonia/pneumonitis. Presence of pulmonary infiltrate(s) on chest x-ray along with a positive BAL specimen/culture, or positive sputum culture, or positive fungal staining/culture, and/or chest CT, and/or transbronchial biopsy specimen to confirm infection and rule out rejection should be present unless strong clinical evidence indicates the need for treatment despite negative cultures. CMV diagnosis requires BAL for cytology or transbronchial biopsy specimen. Diagnosis of Aspergillosis requires fungal histopathology and/or culture of BAL/tissue specimen, bronchoscopic findings consistent with the disease, and possible chest CT[8-15].

6.2 Adverse Event Recording and Reporting

Adverse events are to be collected from the time the transplant begins (time of skin incision) until the completion of the Day30 follow-up evaluation. An adverse event will be followed until resolution or stabilization of the event. All adverse events are to be recorded on the case report forms. The description of the adverse event will include: the date of onset, duration, severity, anticipated or not, the relationship of the event to the study treatment, seriousness, and any treatment that was required.

All (S)AEs will be recorded up to the Day30 follow-up or through hospital discharge if longer than 30 days. All serious adverse events(s) will be documented on the appropriate case report form(s) within 48hours of knowledge.

Per the University of Alberta's HREB, only local SAEs must be reported to the REB if the event is SERIOUS, UNANTICIPATED and considered to be RELATED or POSSIBLY RELATED to the study intervention/device. These SAEs need to be reported within 7 days of their discovery by the study site using the local SAE form.

6.3 Abnormal Clinical & Laboratory Results

Post-transplant clinical and laboratory values for this patient population will inherently be outside of normal ranges. Only test results deemed clinically significant by the study PI will be reported. All laboratory tests will be reviewed by the PI and only those values that are clinically significant will be noted as "CS", signed and dated by the PI.

6.4 Adverse Event Definitions

An adverse event (AE) is any untoward medical occurrence or exacerbation of a pre-existing/baseline medical condition subsequent to the experimental intervention. Adverse events are rated in terms of:

a) **Severity** – where the investigator will rate the severity of the adverse event using the following categories:
- ***Mild:*** The adverse event is transient and/or easily tolerated by the subject.
- ***Moderate:*** The adverse event causes the subject discomfort and interrupts the subject's usual activities.
- ***Severe:*** The adverse event causes considerable interference with the subject's usual activities.

b) **Anticipated or Unanticipated** – where the investigator will assess each adverse event for whether it is anticipated or unanticipated. Anticipated events are defined by our local REB as those events that are "identified in the regulatory documents such as Investigator Brochure or product monograph & occurring within expected frequency estimates, or those identified in REB submission and Letter of Information to participants, or Not related to study intervention, was the result of the natural progression of the person's disease/illness and/or state of health." See Section 6.5 for a list of anticipated adverse events associated.

c) **Device relatedness** – where the investigator will assess the relationship of the AE to the NPV-EVLP or to the standard of care, methods of preservation. The relationship will be assessed using the following categories:
- ***Unrelated:*** There is no relationship between preservation with the NPV-EVLP or with preservation with the standard of care and the adverse event.
- ***Not Likely Related:*** There is a temporal relationship with preservation with the NPV-EVLP or with preservation with the standard of care and the adverse event, but there is not a reasonable causal relationship between the study device and the event.
- ***Possibly Related:*** There is a reasonable relationship with preservation with the NPV-EVLP or with preservation with the standard of care and the adverse event, but the causal relationship is unclear or lacking.

- ***Probably Related:*** It is more likely than not that there is a reasonable causal relationship between preservation with the NPV-EVLP or with preservation with the standard of care and the adverse event.
- ***Definitely Related:*** There is a reasonable causal and temporal relationship between preservation with the NPV-EVLP or with preservation with the standard of care and the adverse event.

6.5 Serious Adverse Events

An adverse event will be categorized as **serious** (SAEs) if, in the view if the Investigator(s), it meets any of the following criteria:

- Results in death;
- It's life-threatening (The subject was at risk of death at the time of the event. It does not refer to an event that hypothetically might have caused death if it were more severe);
- Inpatient hospitalization (>24 hours or overnight admission) or prolongation of existing hospitalization
- Persistent or significant incapacity or substantial disruption of the ability to conduct normal life functions, or
- A congenital anomaly/birth defect, or;
- An important medical event.

Deaths will ONLY be required to be reported as an SAE if they occur within 30days of the date of transplant OR if it is Lung Graft Related and occurred within 6 months of the date of transplant.

The following is a list of SAEs that are *associated* and *anticipated* with double lung transplant procedures which have been documented within the first 30 days following lung transplants, and are therefore **not reportable to the HREB**.

- Acute rejection
- Arrhythmia
- Bleeding (major)
- Hemodynamic instability
- Death
- Fever
- Primary Graft Dysfunction (PGD) as defined by the International Society for Heart & Lung Transplantation (ISHLT) during the first 72 hours post lung transplant
- Respiratory failure
- Graft failure
- Focal or systemic major infection (bacterial, viral, fungal)
- Sepsis
- Emphysema
- Tracheobronchitis/pneumonitis/pneumonia
- Renal dysfunction
- Hyperammonaemia
- Malignancy (post-transplant lymphoproliferative disorder (PTLD)

- Multiple organ failure
- Myocardial infarction
- Neurological dysfunction
- Hepatic dysfunction
- Pancreatitis, peptic ulceration
- Gastro esophageal reflux disease (GERD)
- Aspiration
- Pneumothorax
- Hemothorax
- Pleural bleeding
- Pleural effusion
- Airway anastomotic complications (focal infection, necrosis/dehiscence, stenosis
- Venous thromboembolism (deep venous thrombosis [DVT])
- Pulmonary embolism (PE)
- Pulmonary infarction
- Wound dehiscence
- Organ deemed not transplantable after NPV-EVLP

7 Statistical Plan

7.1 Statistical Methods

All data will be presented in descriptive statistics of all data collected for the duration of the trial. Primary, secondary, and safety endpoints will be compared to a contemporary cohort of patients undergoing double lung transplantation at the University of Alberta during the same time period as the trial. We expect 50-60 patients to be included in the "control" cohort of patients undergoing double lung transplantation with standard criteria donor lungs over the period of this safety trial.

7.2 Subject Population(s) for Analysis

We plan to enroll 12 patients to receive marginal lungs deemed suitable for transplantation using our NPV-EVLP device. We will compare outcomes of these 12 patients to all patients receiving conventional/SOC double lung transplantations using standard criteria lungs over the same time period of the trial. If we expect in a 1-year time frame to enroll 12 patients in this study, that would then include 50-60 patients in the conventional/SOC group over the same period of time as the University of Alberta lung transplant program currently performs 60-70 lung transplants per year.

The data that we will be requesting for SOC/conventional transplants will be provided by the Recipient Transplant Services program in aggregate format. We intend to compare outcome data such as 30Day survival, graft survival at 72hrs, ICU/Hospital LOS, etc. (all of which are collected by the post-transplant team) between the 12 patients enrolled in this study and all other SOC/conventional lung transplants that occur during the same time period as our study enrollment. All SOC transplant data that will be provided to us will be de-identified with only essential outcome data provided for a comparison analysis.

These data points are collected on all transplants that occur at the UAH thus will be available for our comparison analysis.

- Post-Transplant Mechanical Ventilation
- Post-Transplant Length of ICU Stay
- Post-Transplant Length of Hospital Stay
- Cause of Death (overall)
- 1-Year Survival Rate
- Total Length of Survival
- Blood Product Usage Intra-Operative to 48Hrs Post-Op
- Immediate Post-Op Complications such as:
    - Post-Transplant Mechanical Circulatory Support
    - Re-Intubation
    - Renal Replacement Therapy
    - ECMO (Extracorporeal Membrane Oxygenation)
    - Return to OR
    - Tracheostomy
    - Anastomotic
    - Arrhythmias
    - Diaphragm Dysfunction
    - Death

7.3 Significance

As a safety trial, this 12 patient population of patients receiving double lung transplantation after assessment for suitability of marginal donor lungs using the NPV-EVLP is not powered to perform any analysis of significance compared to SOC. The purpose of this trial is to demonstrate safety of the device as it is used to assess and deem suitability of marginal donor lungs over 2-6hours of NPV-EVLP.

7.4 Termination Criteria

An independent Data Safety Monitoring Board (DSMB) will be established by the University of Alberta's Quality Management in Clinical Research (QMCR) to periodically assess the progress of the trial and the safety data and endpoints. The primary function of the DSMB is to monitor the safety of the study at annual intervals. This will include an analysis of all serious adverse events with particular emphasis on Primary, Secondary and Safety Endpoints. The DSMB will make recommendations to the study Investigators regarding continuation, modification or termination of the clinical study. The DSMB will review all data submitted to them by the study Investigators and may request additional information to assist in their decision making process. They will attend scheduled meetings and issue written minutes of their meetings; furthermore, the appointed Chair will be responsible for issuing final written recommendations.

If a termination is recommended by the DSMB, the following are examples of situations that could warrant such a decision:

a) Increased incidence of adverse experiences and/or the severity of such, suggestive of a potential, device-related health hazard;

b) Insufficient subject enrollment;
c) Recurrent protocol non-compliance or deviations;
d) Inaccurate, incomplete, and/or untimely data recording on a recurrent basis.

In the event of a participant death the investigators will stop enrolling patients immediately until the death has been fully reviewed and assessed by the HREB and the study's DSMB. Only when the death has been deemed unrelated to the NPV-EVLP device will enrollment into the trial be reinitiated.

8 Direct Access to Source Data/Documentation

The sponsor, third party monitors, site auditors, and health authority inspectors (from Health Canada and/or the FDA) will be given direct access to source data and documentation (i.e. study subject ICFs, screening log, paper medical charts/records, diagnostics results, computer printouts, (serious) adverse event supporting documentation, recorded data form automated instruments, etc.) for source data verification, provided that subject confidentiality is maintained in accordance with local requirements. Guest access to electronic medical charts/records are not permitted at the University of Alberta Hospital, thus a designated study personnel with study-specific access to study subject data will be present when any electronic data needs to be reviewed/source verified by auditors and monitors.

The investigator must maintain, at all times, the primary records, (i.e. source documents) of each subject's data. Examples of source documents are hospital records, follow-up visit records, examining physician's finding or notes, consultant's written opinion or notes, laboratory reports, device inventory, device label records, and CRFs that are used as the source.

The investigator will maintain a confidential subject identification log that allows the unambiguous identification of each subject.

Per University of Alberta policy, all source data and documentation will be stored for 5 years after the study has ended.

9 Training, Deviations & Monitoring

9.1 Study Staff Training

9.1.1 Procurement of Donor Lungs

Procurement of the marginal donor lungs will be performed by the Human Organ Procurement Exchange (HOPE) program per their SOC organ retrieval protocol. The organs will be brought to the Mazankowski Alberta Heart Institute's OR theatre where trained surgical staff (PI, Co-I or transplant fellows) will instrument the donor lungs onto the NPV-EVLP device. The organs will be perfused and ventilated for a period of 2-6hours. Ongoing evaluation and assessment of the

donor organs will occur until a decision is made, per Section 5.3's 'Post-NPV-EVLP Eligibility Criteria' on whether or not the organs will be transplanted.

9.1.2 Informed Consent at Time of Organ Offer

Written informed consent will be acquired from each recipient who will participate in this study. A letter indicating the purpose of this study will be sent out to all patients currently on the lung transplant waitlist with the study team's contact information included for questions/concerns. Recipients will be screened for eligibility and provide written consent to participate in this study when a suitable set of marginal lungs become available and are matched to that recipient. Since donor lungs can become available at any hour of the day/night, the study coordinator and/or the study Investigator(s) and/or the Pulmonologist involved in the care of the recipient will perform the informed consent process and acquire the recipient's required signature for study consent.

9.1.3 Instrumentation and Perfusion of Donor Lungs

Only medically trained staff members will be instrumenting and perfusing the donor lungs on the NPV-EVLP device. The lead Investigator(s) of this study will provide all required training to transplant fellows/residents/perfusionists/nurses who will be involved the lung perfusion process. All device training will be documented in Training Logs.

The clinical decision on whether or not the donor lungs are acceptable for transplantation post-EVLP will be made by re-assessing the donor lung eligibility post-EVLP and collaborative discussions between the lead Investigator(s), transplant surgeon and the Pulmonologist on-call.

9.2 Protocol Deviations

Throughout the conduct of this study, the Investigator must report to the REB any protocol deviation which:

a) impact the rights, safety or well-being of a research participant; (examples may include: incorrect dose of study medication, enrollment of subject that does not meet the inclusion/exclusion criteria of the protocol, consent violations, subject visits which occur prior to study approval, or after expiry of study approval);
b) jeopardize the study efficacy or the data integrity (examples include ***significant*** variance from the protocol required study procedures, wrong diary or CRF in use, failure of sample storage conditions); OR
c) constitute a breach of privacy.

Deviations from or changes to the protocol to eliminate immediate hazards to the study participants must be reported to the REB within 10 days of their discovery. All other deviations must be reported to the applicable REB within 15 (fifteen) days of their discovery.

Protocol deviations submitted to the REB must include at least the following content:

- A description of the deviation that occurred with an explanation of the circumstances that lead to the deviation and the resulting problem;

- An explanation as to whether or not the deviation compromised the scientific integrity of the study;
- An explanation of whether or not the deviation increased the risk or the possibility of risk for the research subject;
- A description of steps taken or that will be taken to correct / address the problem resulting from the deviation, and;
- A plan for ensuring that a similar deviation does not occur in the future.

9.3 Study Monitoring

This study will be monitored on an ongoing basis by the University of Alberta's QMCR to verify identification, documentation and analysis of adverse event, compliance with the study protocol, and assessment of patient safety at all times within the study.

10 Ethical Considerations

This study will be conducted according to Canadian and international standards of Good Clinical Practice for all studies. Applicable government regulations and University of Alberta research policies and procedures will also be followed.

This protocol and any amendments will be submitted to the University of Alberta HREB for formal approval to conduct the study. The decision of the HREB concerning the conduct of the study will be made in writing to the Investigator. Following initial REB approval of the study application, any changes to the study or approved documentation must be reviewed and approved by REB before implementation, except where necessary to eliminate an immediate hazard(s) to study participants (see Protocol Deviations Section 9.2).

All subjects for this study will be provided a consent form describing this study and providing sufficient information for subjects to make an informed decision about their participation in this study. This consent form will be submitted with the protocol for review and approval by the HREB. The formal consent of a subject, using the HREB-approved consent form, will be obtained before that subject is submitted to any study procedure. This consent form must be signed by the subject or legally acceptable surrogate, and the investigator-designated research professional obtaining the consent.

11 Data Handling and Record Keeping

11.1 Completing, Signing and Archiving Case Report Forms

The site principle investigator must keep a separate subject identification log indicating study subject ID numbers, names, and dates of birth to allow unambiguous identification of each individual enrolled in the study. A note will be made in each subject's medical charts that he/she is participating in this clinical trial. This note has to be signed and dated by the PI or the approved designee at time of enrollment.

All data to be collected for study subjects will be recorded on donor and recipient study CRFs. The CRFs will be completed legibly in ink or electronically with reasons given for missing values for protocol required data. All CRFs must be kept in good order and updated so they always reflect the latest observations on the subjects participating in the study.

The investigator (or assigned designee) will sign the appropriate pages of each CRF. Any corrections/changes to the study data will be made in a manner that does not obscure the original entry (ie. striking a single line through erroneous data and clearly entering the correct data; ex: ~~wrong data~~ right data) and will be dated and initialed in blue or black ink on the day that the change is made by the investigator or assigned designee.

11.2 Data Management and Archiving

The site Principle Investigator and/or assigned designee will be responsible for the processing and quality control of the data. All study-related documents will be maintained by the Investigator for 25 years following study completion per Health Canada regulations on clinical research. Records will be retained in a secure, dry location controlled by the institution.

12 Abbreviations & Definitions

CRF	Case Report Form
CSA	Canadian Standards Association
DCD	Donor after Cardiocirculatory Death
DSMB	Data Safety Monitoring Board
EVLP	Ex-vivo Lung Perfusion
FEV1	Forced Expiratory Volume in 1second
FiO_2	Fraction of Inspired Oxygen
GCP	Good Clinical Practice
HREB	Health Research Ethics Board
ICH	International Conference on Harmonization
ISHLT	International Society for Heart and Lung Transplantation
ISO	International Organization of Standardization
NDD	Donor after Neurological Death
NPV-EVLP	Negative Pressure Ventilation Ex-vivo Lung Perfusion
PaO_2	Partial Pressure of Oxygen
PGD	Primary Graft Dysfunction

SOC Standard of Care

QMCR Quality Management in Clinical Research

UAH University of Alberta Hospital

13 References

1. Christie JD, Edwards LB, Kucheryavaya AY, Benden C, Dobbels F, Kirk R, Rahmel AO, Stehlik J, Hertz MI. The Registry of the International Society for Heart and Lung Transplantation: Twenty-eighth Adult Lung and Heart-Lung Transplant Report--2011. *J Heart Lung Transplant.* 2011;30(10):1104-1122.
2. Cypel M, Keshavjee S. Extracorporeal lung perfusion. *Curr Opin Organ Transplant.* 2011;16(5):469-475.
3. Cooper JD, Pearson FG, Patterson GA, Todd TR, Ginsberg RJ, Goldberg M, DeMajo WA. Technique of successful lung transplantation in humans. *J Thorac Cardiovasc Surg.* 1987;93(2):173-181.
4. Patterson GA, Cooper JD, Dark JH, Jones MT. Experimental and clinical double lung transplantation. *J Thorac Cardiovasc Surg.* 1988;95(1):70-74.
5. Steen S, Sjoberg T, Pierre L, Liao Q, Eriksson L, Algotsson L. Transplantation of lungs from a non-heart-beating donor. *Lancet.* 2001;357(9259):825-829.
6. Cypel M, Yeung JC, Hirayama S, Rubacha M, Fischer S, Anraku M, Sato M, Harwood S, Pierre A, Waddell TK, de Perrot M, Liu M, Keshavjee S. Technique for prolonged normothermic ex vivo lung perfusion. *J Heart Lung Transplant.* 2008;27(12):1319-1325.
7. Cypel M, Yeung JC, Liu M, Anraku M, Chen F, Karolak W, Sato M, Laratta J, Azad S, Madonik M, Chow CW, Chaparro C, Hutcheon M, Singer LG, Slutsky AS, Yasufuku K, de Perrot M,

Pierre AF, Waddell TK, Keshavjee S. Normothermic ex vivo lung perfusion in clinical lung transplantation. *N Engl J Med.* 2011;364(15):1431-1440.

8. Christie, JD, Carby M, Bag R, Corris P, Hertz M, Weill D. Report of the ISHLT Working Group on Primary Lung Graft Dysfunction: Part II. Definitions. J Heart Lung Transplant 2005;24:1454-9.
9. Paradis IL, Williams P. Infection after lung transplantation. SeminRespir Infect 1993;8:207-15.
10. Maurer JR, Tullis E, Grossman RF, Vellend H, Winton TL, Patterson GA. Infectious complications following isolated lung transplantation. Chest 1992;101:1056-9.
11. Horvath J, Dummer S, Lloyd J, Walker B, Merrill WH, Frist WH. Infection in the transplanted and native lung after single lung transplantation. Chest 1993;104:681-5.
12. Zamora MR. Cytomegalovirus and lung transplantation. Am J Transplant 2004;4:1219-26.
13. Singh N, Husain S. Aspergillus infections after lung transplantation: clinical differences in type of transplant and implications for management. J Heart Lung Transplant 2003;22:258-65.
14. Silveira FP, Husain S. Fungal infections in lung transplant recipients. CurrOpinPulm Med 2008;14:211-8.
15. Lau CL, Patterson GA, Palmer SM. Critical care aspects of lung transplantation. J Intensive Care Med 2004;19:83-104.
16. Kotloff, RM, Ahya, VN. Medical complications of lung transplantation. EurRespir J 2004;23:334-42.
