# Supplementary material for: Clinical transplantation using negative pressure ventilation ex situ lung perfusion with extended criteria donor lungs
Source: Nat Commun. 2020 Nov 13;11:5765. doi: 10.1038/s41467-020-19581-4 (PMC7666579; doi:10.1038/s41467-020-19581-4)
Supplement: Supplementary file 3 — Reporting Summary [file 41467_2020_19581_MOESM3_ESM.pdf]

nature research

Corresponding author(s): Jayan Nagendran

Last updated by author(s): Sep 22, 2020

Reporting Summary

Nature Research wishes to improve the reproducibility of the work that we publish. This form provides structure for consistency and transparency in reporting. For further information on Nature Research policies, see our Editorial Policies and the Editorial Policy Checklist.

Statistics

For all statistical analyses, confirm that the following items are present in the figure legend, table legend, main text, or Methods section.

n/a	Confirmed	
☐	☒	The exact sample size (*n*) for each experimental group/condition, given as a discrete number and unit of measurement
☒	☐	A statement on whether measurements were taken from distinct samples or whether the same sample was measured repeatedly
☒	☐	The statistical test(s) used AND whether they are one- or two-sided<br>*Only common tests should be described solely by name; describe more complex techniques in the Methods section.*
☒	☐	A description of all covariates tested
☒	☐	A description of any assumptions or corrections, such as tests of normality and adjustment for multiple comparisons
☐	☒	A full description of the statistical parameters including central tendency (e.g. means) or other basic estimates (e.g. regression coefficient) AND variation (e.g. standard deviation) or associated estimates of uncertainty (e.g. confidence intervals)
☒	☐	For null hypothesis testing, the test statistic (e.g. *F*, *t*, *r*) with confidence intervals, effect sizes, degrees of freedom and *P* value noted<br>*Give P values as exact values whenever suitable.*
☒	☐	For Bayesian analysis, information on the choice of priors and Markov chain Monte Carlo settings
☒	☐	For hierarchical and complex designs, identification of the appropriate level for tests and full reporting of outcomes
☒	☐	Estimates of effect sizes (e.g. Cohen's *d*, Pearson's *r*), indicating how they were calculated

Our web collection on statistics for biologists contains articles on many of the points above.

Software and code

Policy information about availability of computer code

Data collection	Data was collected on Microsoft Excel Version 16.16.25 (Volume License 2016).
Data analysis	All analyses were performed on STATA 15 (StataCorp LLC, College Station, Texas).

For manuscripts utilizing custom algorithms or software that are central to the research but not yet described in published literature, software must be made available to editors and reviewers. We strongly encourage code deposition in a community repository (e.g. GitHub). See the Nature Research guidelines for submitting code & software for further information.

Data

Policy information about availability of data

All manuscripts must include a data availability statement. This statement should provide the following information, where applicable:

- Accession codes, unique identifiers, or web links for publicly available datasets
- A list of figures that have associated raw data
- A description of any restrictions on data availability

The data that support the findings of this study are available on request from the corresponding author, Jayan Nagendran. The data are not publicly available due to the University of Alberta Human Ethics Research Board patient confidentiality restrictions as they contain information that could compromise research participant privacy and consent.

Field-specific reporting

Please select the one below that is the best fit for your research. If you are not sure, read the appropriate sections before making your selection.

- [x] Life sciences
- [ ] Behavioural & social sciences
- [ ] Ecological, evolutionary & environmental sciences

For a reference copy of the document with all sections, see nature.com/documents/nr-reporting-summary-flat.pdf

Life sciences study design

All studies must disclose on these points even when the disclosure is negative.

Sample size	Lungs deemed marginal, based on standard lung donor criteria that met the study eligibility criteria, were assessed on the NPV-ESLP device to determine suitability for lung transplantation. Objective assessment of quality was be made while the lungs are on the device based on pre-determined functional parameters of lung physiology. Enrollment was concluded once a pre-specified total of 12 sets of lungs were transplanted after using the device. This number was determined in conjunction with the institutional ethics review board and is similar to other safety trials within the field (Lancet 2012; 380: 1851–58).
Data exclusions	All 12 sets of lungs that underwent Negative Pressure Ventilation Ex Situ Lung Perfusion were included in the study. The were no organs or patients excluded.
Replication	This study represents a first in-human clinical trial, as such there is not replication of human results. These results support the further validation of clinical Negative Pressure Ventilation Ex Situ Lung Perfusion by other investigators.
Randomization	As lungs used in this study were based on the submitted inclusion criteria for extended criteria donor lungs, this cannot be a randomized clinical trial.
Blinding	There was no blinding of investigator or patient as informed consent to use the technology was required prior to enrolling patients into the trail when an extended criteria donor lung offer was identified.

Reporting for specific materials, systems and methods

We require information from authors about some types of materials, experimental systems and methods used in many studies. Here, indicate whether each material, system or method listed is relevant to your study. If you are not sure if a list item applies to your research, read the appropriate section before selecting a response.

Materials & experimental systems

n/a	Involved in the study	
x		Antibodies
x		Eukaryotic cell lines
x		Palaeontology and archaeology
x		Animals and other organisms
	x	Human research participants
	x	Clinical data
x		Dual use research of concern

Methods

n/a	Involved in the study	
x		ChIP-seq
x		Flow cytometry
x		MRI-based neuroimaging

Human research participants

Policy information about studies involving human research participants

Population characteristics	Lungs were obtained from donors aged at least 16 years who's family members had provided appropriate consent for the donation of their organs. Donor inclusion criteria included any of the following: P:F ratio < 300mmHg, Maastricht III or IV deceased from cardiac death donors (DCD), > 10 units of blood transfusion, expected cold ischemic time > 6 hours, or donor age > 55 years old. Donor exclusion criteria included established pneumonia, severe mechanical lung injury, and documented infectious disease in the donor including HIV, Hepatitis, HTLV, or syphilis. Recipient inclusion criteria included all patients aged >18 years old who are accepted on the transplant institute's waitlist for bilateral transplantation who provided prospective written consent to being part of the trial. Recipient exclusion criteria included multi-organ recipients or re-transplants, hemodialysis or severe chronic renal dysfunction, concurrent cardiac procedure, or pre-operative mechanical circulatory support or mechanical ventilation (excluding CPAP or BiPAP).<br><br>The patients undergoing transplantation had a mean age of 58 ± 3 years, height 1.70 ± 0.02 m, and weight 76.1 ±3.9 kg. Indications for transplantation included emphysema (42%), talcosis (17%), IPF (17%), alpha-1-anti-trypsin deficiency (8%), cystic fibrosis (8%), and NSIP (8%).
Recruitment	Lungs were obtained from donors aged at least 16 years who's family members had provided appropriate consent for the donation of their organs. All patients aged at least 18 years listed on the University of Alberta Lung Transplantation recipient wait list that met the inclusion criteria were approached for recruitment if an an extended criteria donor lung offer became available that would match the recipient size and histo-compatibility. Informed consent for recruitment in the trial was obtained at the time of listing and again at the time of transplantation. Donor allocation is dependent on Canadian guidelines on lung allocation scoring which determines priority of recipients. The limitations of current Canadian lung allocation algorithms may bias the results to selecting specific recipient populations.
Ethics oversight	The study was approved by the institutional ethics review board at the University of Alberta [REB Approval: Pro00070552, (July 27, 2019)].

Note that full information on the approval of the study protocol must also be provided in the manuscript.

Clinical data

Policy information about clinical studies

All manuscripts should comply with the ICMJEguidelines for publication of clinical research and a completedCONSORT checklist must be included with all submissions.

Clinical trial registration	Clinicaltrials.gov: NCT03293043
Study protocol	The trial protocol is attached in the supplementary material.
Data collection	Recruitment of data officially began on October 11, 2018, and data collection was completed July 15, 2019. Trial data was recorded at scheduled trial follow-up appointments according to the trial protocol. Perfusion data was recorded at the University of Alberta Hospital. Lung donor data was recorded at site of organ procurement. Data was stored on institutional computers, using Microsoft Excel Version 16.16.25 (Volume License 2016).
Outcomes	The primary endpoint was a composite of survival to 30 days post-transplant and absence of primary graft dysfunction grade 3 (PGD3) within 72 hours after transplantation. Secondary outcomes included PGD scores at 0, 24, 48, and 72 hours post-transplantation, intensive care unit length of stay, hospital length of stay, duration of invasive mechanical ventilation post-transplantation, and survival to one-year post-transplantation.
